# Amylene

**Published:** 1858-01

**Authors:** 


### ARTICLE IV.

### *Amylene.* Its Preparation, History and Use.

The new anæsthetic introduced to the notice of the medical profession by Dr. Snow, of London, has been now long enough before the public to enable us to form a definite opinion as to its merits, absolute and comparative. We propose, therefore,to give a brief history of the introduction of this agent, and to state its present position in the scientific world.

Every one who has been present in a distillery in which alcohol is made from whiskey, must have been conscious of a peculiar pungent odor, altogether distinct from that of the alcohol itself. This proceeds from the so-called "oil of whiskey," a liquid which separates from the alcohol during the process of distillation. It is usually milky, owing to the imperfect admixture of the substances which compose it.

If this liquid be subjected to distillation, there passes over, first, a clear colorless liquid which possesses the pungent odor in question, in a very slight degree. Gradually the heat rises, and the operator sees a liquid, in oily drops, passing down his tube and gathering in his receiver. It emits most pungent, irritating vapors, and few lungs can receive them without being excited to fits of violent coughing. This is fusel oil, or hydrated oxyd of amyl.

If to fusel oil, so prepared, chloride of zinc be added, and heat applied, a homogeneous liquid is formed, which, at about 266° F. begins to distil. The product thus obtained, when redistilled, goes over fractionally, a portion volatilizing as low as 140° F., and the boiling point of the liquid ultimately rising to 570° F. The most volatile parts are now agitated with concentrated sulphuric acid, when a colorless and very mobile liquid rises to the surface. This is *amylene*, first discovered and described in 1844, by M. Balard, professor of chemistry at the Faculty of Science, of Paris. It must not be confounded with another substance to which M. Auguste Cahours gave the same name five years before, but which is now called paramylene.

Amylene is of very low specific gravity; according to Dr. Snow, 0.659 at 56° F. Its boiling point is 102° F., and the density of its vapor, 2.45. It is composed of ten atoms of carbon and ten of hydrogen, and bears the same relation to fusel oil, or amylic alcohol, that olefiant gas, or ethylene bears to common alcohol. It burns with a brilliant, white flame, is soluble in alcohol and ether in all proportions, but sparingly soluble in water. Dr. Snow considers its odor rather pleasant, and compares it to naptha; others say it reminds one sometimes of coal tar, sometimes of bad malt, and they consider it utterly unbearable.

The first experiments in the use of amylene on the human subject, were made by Dr. Snow, on the 10th of November, 1856, after having previously informed himself of its action by numerous trials of it upon the lower animals. The subjects were two patients who had to undergo the operation for the extraction of teeth. In these the action of the anæsthetic was not entirely satisfactory. On the 4th of December, the same gentleman employed it upon four new patients with complete success. After this it was used by different surgeons, in grave cases, until by the close of January, 1857, no fewer than sixty-nine operations had been performed in England, by its aid.

In Paris, amylene was first employed in the early part of February, in the wards under the charge of M. Aran, and here, also, the patients first operated upon, had come to get teeth extracted. The experiment was a failure, twenty minutes having been consumed in inhalation, without the production of anæsthesia. The failure was attributed to the instrument used, which did not allow sufficient amylene to be inhaled. In a subsequent trial with another inhaling apparatus, the success was complete.

About the same time, M. Giraldes, surgeon to the Foundling Hospital, made some experiments upon his little patients, and on the 4th of March reported favorably upon its action. Attention being now strongly attracted to the new anæsthetic, the Academy of Medicine, at Paris, appointed a committee consisting of MM. Robert, Velpeau, and Malgaigne, to examine the matter. They did so and reported to the Academy on the 12th of last May.

Meanwhile, amylene, which had hitherto been considered harmless, had killed a man in England. The accident happened in the hands of Dr. Snow, who was administering the agent for the 144th time. Mr. Ferguson was operating upon the patient for fistula in ano. There were some remarkable points about the case. Everything seemed to be going on well, when the pulse suddenly fluttered and failed. Strangely enough, however, the respiration continued natural, and the anæsthesia appeared imperfect, since the patient moved his features and limbs a little, as though about to awake. In two or three minutes, however, the insensibility evidently deepened, the breathing getting slower. Presently the countenance became livid, and the respiration gasping. Marshall Hall's "ready method" was now tried, with the effect of causing an influx of air into the lungs. All efforts, however, were unavailing, the patient died.

The post-mortem examination revealed nothing. There was some dilatation of the right ventricle ; some thickening of the walls of the left. The lungs were large and did not collapse, so that Dr. Snow thinks they were emphysematous, although no large cells could be found on the surface.

Amylene is far less soluble in the animal fluids than chloroform. Dr. Snow calculates, that in the third degree of narcotism from the use of this agent, there cannot be more than three minims circulating in the blood. Still the dose must be much larger than that of chloroform or even ether, because owing to the high tension of its vapor, and its great insolubility, the largest proportion of what is inhaled is expelled from the lungs without entering the circulation. Besides, the small quantity which is absorbed, is speedily expelled again, so that the anæsthesia is of short duration, and the inhalation must be very frequently repeated to keep up the impression; hence the quantity of it consumed is necessarily very large. The vapor must also be concentrated. The air inhaled must contain at least fifteen per cent. of this agent, in order that it may produce the third degree of narcotism, or that stage in which consciousness and voluntary motion are entirely suspended. It must be given at the rate of rather more than a fluid drachm a minute, when it will, usually, produce insensibility in three minutes, according to Dr. Snow. It cannot be administered without the inhaler.

It produces anæsthesia without the profound coma attendant upon the action of ether and chloroform. In some instances partial or complete consciousness remains, though the sensibility to pain is deadened. The circulation is accelerated, the countenance flushed, the head thrown back, and the limbs sometimes stiffened and extended. The latter symptom is said by Dr. Snow, to be a rare accompaniment of the administration of amylene. Snow and Robert both agree in the statement, that relaxation of the muscles does not occur from the use of this agent, but M. Tourde says that relaxation can be produced if the anæsthesia is kept up a sufficient length of time. It does not irritate the air passages, nor does it, like chloroform, induce nausea and vomiting. Its influence, as has already been said, is very fugacious, generally ceasing the moment the inhaler is withdrawn. Furthermore, it does not leave that sense of discomfort which occasionally follows the use of chloroform.

The report alluded to does not propose amylene as a substitute for chloroform in all cases, but only in those in which the operation is to be of short duration.

Velpeau, though signing the report with his colleagues, stated to the Academy that he was not a great admirer of amylene. He said he had tried it, but found it inferior to chloroform. "I find fault with it," says he, "for its detestable smell, as inconvenient for the assistants as for the patient, the little certainty and constancy of its action, the too short continuance of its effects, and the necessity of using a special inhaler for it." He thought the danger of chloroform greatly overrated, and that it was not more dangerous than amylene, if carefully administered.

Before closing this very brief notice of a substance which has already called forth such voluminous articles, it is proper to state that Dr. Kadlburger relates the results of the employment of amylene in seventy-two cases of tooth extraction. His paper appeared in the Wien Wochenschrift, No. 19. He uses no inhaler, but simply covers the mouth and nose with a sponge saturated with the anæsthetic. He states that the amylene he employs has the odor of over ripe pears, and is not at all irritating to the respiratory organs. He accomplishes anæsthesia in a minute or a minute and a half, the signal of its establishment being the dropping of the uplifted arm, as if fatigued. No abnormal muscular movement or closure of the jaw takes place. The patient is docile, and if he feels any pain at all, feels it very slightly.

